# Rehabilitation Practice and Association with Outcomes in Critically Ill Invasively Ventilated Patients (REPOrt): a protocol for an international multicenter observational study

**DOI:** 10.62675/2965-2774.20260463

**Published:** 2026-09-02

**Authors:** Denise Battaglini, Rik Gosselink, Sabrine N. T. Hemmes, Carol L. Hodgson, Ricardo Kenji Nawa, Irene Schiavetti, Ary Serpa, Gentle S. Shrestha, Marcus J. Schultz, REPOrt investigators, Report Steering Committee Members, Denise Battaglini, Rik Gosselink, Sabrine N.T. Hemmes, Carol L. Hodgson, Ricardo Kenji Nawa, Ary Serpa, Irene Schiavetti, Gentle S. Shrestha, Marcus J. Schultz, Report Investigators, Mohammad Jhahidul Alam, Zahedul Alam, Abdulrahman Al-Fares, Sara Albertinetti, Loui Al-Husinat, Lilian Ayako Henriques Luciani, Joao Pedro Alves, Ioannis Andrianopoulos, Martin Angulo, Massimo Antonelli, Irene Aragao, Ricardo Arriagada, Kaniye Aydın, Müge Aydoğdu, Natalia Barrel Cota, Denise Battaglini, Anurag Bajracharya, Nisha Bhandari, Sabin Bhandari, Sunil Bhandari, Luca Bastia, Mustafa Kemal Bayar, Giacomo Bellani, Gabriele Bera, Pushkar Bishwokarma, Maria Maddalena Bitonto, Maria Grazia Bocci, Nazlihan Boyaci, Brisa Bribiesca, Iacopo Cappellini, Vinicio Danzi, Sujan Dhakal, Marcio Luiz Ferreira de Camillis, Rafael de Moraes Ianotti, Maycon de Moura Reboredo, Silvia De Rosa, Lígia dos Santos Roceto Ratti, Subhal B. Dixit, Lerzan Dogan, Katia Donadello, Antonio Esquinas, Valentina Fassone, Susana M. Fernandes, Leyla Ferlicolak, Angelo Giacomucci, Rupesh Gami, Basanta Gauli, Angelo Giacomucci, Ernestina Gomes, Caroline Gomes Mol, Saint-Clair Gomes Bernardes, Carolline Gonzaga Ferreira, Rik Gosselink, Leonardo Gottin, Domenico Luca Grieco, Agnese Grilli, Matjaz Groznik, Francesca Gualdi, Adi Hadzibegovic, Sabrine N. T. Hemmes, Carol L. Hodgson, Farhana Karachi, Kiran Karki, Roman Karki, Ahmet Safa Kaynar, Paula Fernandes, Drin Kamishi, Hiroshi Koyama, Kamil İnci, John Laffey, Sergio Lassola, Gianluigi Li Bassi, Livia Lovelace, Lisa Lubuschagne, Ines Machado Vaz, Vinícius Zacarias Maldaner da Silva, Jihad Mallat, Raffaele Mandarano, Roberto Martinez-Alejos, Joan Daniel Martì Romeu, Elshiekh Mihammed, Sachi Miyashita, Ícaro Montenegro Marques, Seyed Mohammadreza Hashemian, Rommer Alex Ortega Martinez, El Sheikh Othman Mohamed, El Sheikh Othman Mohamed, Federico Moro, Bijay KC, Ricardo Kenji Nawa, Filip Naumovski, Daniela Nisticò, Prastab Parajuli, Fernanda Maia Passos Garrido, Shreeya Pathak, Nicolò Antonino Patroniti, Gaetano Perchiazzi, Wellington Pereira Yamaguti, Ornella Piazza, Lise Piquilloud, Ricardo Pinho, Sangina Ranjit, Nitendra Raj Bajracharya, Rodney Recalde, Margarida Ribeiro, Ankit Rimal, Raissa Maria Rocha dos Santos, Roberta Mazzali Rodolfo Biscaro, Ana Rodrigues, Abobakr Saleh, Román Morales Sánchez, Mariana S. Santos, Simone Santosuosso, Irene Schiavetti, Jordi Serras, Débora Stripari Schujmann Nogueira, Rishav Sharma, Ary Serpa, Gentle S. Shrestha, Marcus J. Schultz, Milton Santillan, Adriana Silva, Lucinara Martins Silva Tallarico, Andrej Sribar, Subarna Thapa Chhetri, Eyob Tesfaye, Laura Trevino, David Thomson, Ahmet Uysal, Katerina Vaporidi, Sutharshan Vengadasalam, Kumar Vishal, Jacob Wittenstein, Erdem Yalçınkaya, Cassio Luis Zandonai, José J. Zaragoza, Mateusz Zawadka

**Affiliations:** 1 University of Genoa Department of Surgical Sciences and Integrated Diagnostics Genoa Italy Department of Surgical Sciences and Integrated Diagnostics, University of Genoa - Genoa, Italy.; 2 University of Leuven Faculty of Movement and Rehabilitation Sciences Department of Rehabilitation Sciences Leuven Belgium Department of Rehabilitation Sciences, Faculty of Movement and Rehabilitation Sciences, University of Leuven - Leuven, Belgium.; 3 Amsterdam University Medical Centers Department of Anaesthesiology Amsterdam Netherlands Department of Anaesthesiology, Amsterdam University Medical Centers - Amsterdam, The Netherlands.; 4 Monash University Australian and New Zealand Intensive Care Research Centre Melbourne Australia Australian and New Zealand Intensive Care Research Centre, Monash University - Melbourne, Australia.; 5 Hospital Israelita Albert Einstein Department of Critical Care Medicine São Paulo SP Brazil Department of Critical Care Medicine, Hospital Israelita Albert Einstein - São Paulo (SP), Brazil.; 6 University of Genoa Department of Health Sciences Genoa Italy Department of Health Sciences, University of Genoa - Genoa, Italy.; 7 Tribhuvan University Teaching Hospita Maharajgunj Kathmandu Nepal Tribhuvan University Teaching Hospita - Maharajgunj, Kathmandu, Nepal.; 8 Medical University Wien Division of Cardiothoracic and Vascular Anaesthesia & Critical Care Medicine Department of Anaesthesia, General Intensive Care and Pain Management Vienna Austria Department of Anaesthesia, General Intensive Care and Pain Management, Division of Cardiothoracic and Vascular Anaesthesia & Critical Care Medicine, Medical University Wien - Vienna, Austria.

**Keywords:** Critical care, Intensive care, Invasive ventilation, Rehabilitation, Physiotherapy, Length of stay, Morbidity, Mortality

## Abstract

**Background:**

Although interventions such as early mobilization, physical activity, and respiratory physiotherapy have demonstrated benefits in critically ill patients, they remain inconsistently implemented in routine practice. Patients requiring invasive mechanical ventilation are particularly vulnerable to intensive care unit-acquired weakness, prolonged disability, and adverse long-term outcomes. The primary objective of the *Rehabilitation Practice and Association with Outcomes in Critically Ill Invasively Ventilated Patients* (REPOrt) study is to describe the prevalence and characteristics of rehabilitation practices in invasively ventilated intensive care unit patients worldwide. Secondary, exploratory objectives include assessing associations between rehabilitation exposure and selected clinical and functional outcomes.

**Methods:**

REPOrt is an investigator-initiated, international, multicenter, prospective observational study designed to describe rehabilitation practices in adult intensive care unit patients. Eligible patients are adults receiving invasive mechanical ventilation for at least 48 hours in a participating intensive care unit. The primary objective is to determine the proportion of patients receiving at least one rehabilitation intervention during the intensive care unit stay, including mobilization (active or passive) and/or respiratory physiotherapy (active or passive). Secondary objectives include describing the characteristics of rehabilitation practices (type, timing, duration, and intensity); evaluating clinical outcomes such as intensive care unit and hospital mortality, intensive care unit and hospital length of stay, duration of invasive mechanical ventilation, and intensive care unit-related complications; and assessing quality of life and functional performance 28 days after intensive care unit discharge. REPOrt consists of three modules. The mandatory module collects patient demographics, baseline characteristics, rehabilitation exposure, and short-term outcomes. Two optional modules complement the core dataset: one captures detailed information on specific rehabilitation interventions, including physical exercises, respiratory therapy, dysphagia management, occupational therapy, and cognitive support; the other focuses on extended follow-up, assessing quality of life and functional status. Each participating center will enroll patients over 120 days, with an expected inclusion of 20 - 40 patients per site. At least 60 centers are anticipated to participate, for an expected total sample size of approximately 2,400 patients.

## INTRODUCTION

Critically ill patients are at high risk of developing intensive care unit (ICU)-acquired weakness (ICUAW), defined as clinically detectable weakness without any other plausible explanation.^(1)^ Its incidence is substantial, affecting up to 40% of patients,^(2-8)^ and it is strongly associated with both morbidity and mortality.^(9)^ Among the contributing factors, invasive mechanical ventilation plays a key role as it accelerates the loss of skeletal muscle mass, thereby promoting the development of ICUAW.^(10-13)^

Mobilization has emerged as an effective rehabilitation strategy that improves both short- and long-term outcomes, including reduced ICU- and hospital-length of stay and enhanced functional recovery.^(14-16)^ Evidence consistently demonstrates these benefits, underscoring the important role of rehabilitation professionals in the ICU.^(17,18)^ Timely initiation of rehabilitation is particularly relevant, as it decreases ventilator dependence and hospital-acquired complications, with lasting effects on quality of life and cognitive function after discharge.^(19,20)^ Nevertheless, despite its proven benefits, rehabilitation remains underutilized in many ICUs, likely due to resource and staffing limitations,^(21)^ and it remains uncertain how best to perform rehabilitation in terms of timing, type, intensity, and frequency.

A wide range of rehabilitation interventions can be applied to invasively ventilated critically ill patients, including mobilization, active and passive exercise, respiratory physiotherapy, dysphagia training, occupational therapy, physical activity, and cognitive support.^(22-25)^ These interventions can be broadly categorized into passive approaches, such as neuromuscular electrical stimulation, and active approaches, such as muscle training and ambulation. Understanding each patient's condition and specifying how rehabilitation is delivered, including the type of intervention, its duration, frequency, and intensity, is crucial for tailoring safe and effective therapy.^(16,25-32)^

We designed the *Rehabilitation Practice and Association with Outcomes in Critically Ill Invasively Ventilated Patients* (REPOrt) study as a prospective, international, multicenter, observational investigation. REPOrt aims to characterize real-world rehabilitation practices, focusing on mobilization and respiratory physiotherapy across a large, diverse and worldwide sample, by including high-, middle- and low-income countries, and different types of ICU and hospital. Study objectives are hierarchically structured. The primary objective is descriptive and aims to quantify the prevalence and characteristics of rehabilitation practices in invasively ventilated ICU patients. Secondary objectives are exploratory and aim to investigate associations between rehabilitation exposure and clinical, functional, and patient-centered outcomes.

We hypothesize that rehabilitation practice varies substantially across geo-economic regions and clinical settings, and that such variability may be associated with differences in important patient outcomes, including ICU and hospital mortality, ICU and hospital length of stay, duration of invasive mechanical ventilation, and complications occurring during the ICU stay. Furthermore, we hypothesize that rehabilitation exposure may influence post-discharge functional status and quality of life.

## METHODS

### Study design and setting

REPOrt is a prospective, international, multicenter, observational cohort study of critically ill, invasively ventilated patients that uses three modules, with centers able to participate contingent on local capabilities. REPOrt was approved by the Institutional Review Board (IRB) of Liguria Territory on March 17^th^, 2025 (419/2024) and is registered at clinicaltrials.gov (NCT07093125). The study enrolment is planned to start in the autumn of 2025, with a 120-day enrolment period at each participating center. Potentially interested ICUs will be identified via email contact and personal meetings at congresses and symposia. Intensive care units that take care of adult invasively ventilated critically ill patients are eligible for participation in REPOrt. If a hospital has more than one ICU, each ICU can participate as long as the patients cared for are adults, as defined by local legal age criteria. The Research Office of IRCCS *Azienda Ospedaliera Metropolitana*, *Ospedale Policlinico San Martino*, Genova, Italy, handles the list of participating centers, as well as the list of primary investigators from each ICU. Where applicable, centers should submit the study protocol to their local IRB for approval. Informed Consent must be obtained in accordance with local regulations in each country. More information on ethical considerations is provided in Item 1S (Supplementary Material). The study will follow the Strengthening the Reporting of Observational Studies in Epidemiology (STROBE) guidelines.

### Patients

Patients are eligible for participation if admitted to a participating ICU; and receiving invasive ventilation for at least 48 hours. Patients in whom withdrawal of life-sustaining treatment occurs within the first 48 hours of invasive mechanical ventilation, prior to meeting eligibility criteria, are excluded. Withdrawal of life-sustaining treatment occurring after cohort inclusion does not lead to exclusion.

### Endpoints

For the primary descriptive endpoint, rehabilitation exposure is defined as a composite outcome: receipt of any rehabilitation intervention during the ICU stay, including mobilization and/or respiratory physiotherapy, whether passive or active.

REPOrt has study endpoints that differ according to the three modules. These include the proportion of patients receiving at least one rehabilitation intervention (primary endpoint), granular data on rehabilitative interventions (including type of mobilization, type of respiratory physiotherapy, timing, duration, and intensity), ICU mortality, ICU length of stay, Invasive mechanical ventilation duration during ICU stay, ICU complications, hospital mortality, and hospital length of stay. Additional secondary endpoints include quality of life and functional performance at 28 days after ICU discharge, as well as mortality at that time.

### Variable definitions

Rehabilitation interventions are categorized according to patient participation (passive vs active) and therapeutic domain (mobilization vs respiratory physiotherapy), as summarized in table 1.

**Table 1:** Classification of rehabilitation interventions according to patient participation and therapeutic domain

Domain	Passive interventions (no voluntary effort)	Active interventions (voluntary participation)
Mobilization	Passive exercise; stretching; passive cycling; continuous passive motion; splinting; neuromuscular electrical stimulation	Active exercises; functional movements; sitting, standing, transfers, ambulation (e.g., using intensive care unit mobility scale)
Respiratory physiotherapy	Manual/ mechanical techniques; positioning techniques (e.g., postural drainage, side-lying, prone); passive lung expansion (e.g., manual hyperinflation, mechanical insufflation-exsufflation, continuous positive airway pressure/non-invasive ventilation cycles); airway clearance support (e.g., suctioning); humidification therapy; passive neuromuscular facilitation (e.g., diaphragmatic facilitation techniques)	Breathing exercises (e.g., diaphragmatic breathing, pursed-lip breathing); active airway clearance techniques (e.g., Active Cycle of Breathing Techniques, Forced Expiratory Technique, positive expiratory pressure); lung expansion exercises (e.g., incentive spirometry, inspiratory muscle training); functional respiratory training (e.g., respiratory muscle endurance); physical activity with respiratory focus (e.g., cycle ergometer with respiratory cues); active neuromuscular techniques

Detailed intervention options and data entry fields are available in the electronic case report form. Individual intervention types are recorded in detail within the electronic case report form; the table summarizes conceptual categories for interpretability.

Passive mobilization includes interventions delivered without the patient's active voluntary muscle contraction. These interventions are therapist- or device-driven and aim to preserve joint mobility, muscle length, or circulation. In the electronic case report form (eCRF), passive mobilization includes passive exercises, stretching, passive cycling, continuous passive motion, splinting, and neuromuscular electrical stimulation.

Active mobilization includes interventions that require active, voluntary patient participation, with or without assistance, such as active exercises, functional movements, transfers, standing, and ambulation (captured separately in the eCRF).

Passive respiratory physiotherapy refers to respiratory interventions delivered without active patient effort, including positioning strategies, passive lung expansion techniques, airway clearance support, humidification therapy, and neuromuscular facilitation techniques applied without voluntary activation.

Active respiratory physiotherapy refers to respiratory interventions requiring active patient participation, including breathing exercises, active airway clearance techniques, lung expansion exercises, functional respiratory training, and neuromuscular techniques performed with voluntary control.

### Data sources and measurements

Data collected in the study will be captured on an eCRF developed in the REDCap Platform, hosted by the IRCCS *Azienda Ospedaliera Metropolitana*, *Ospedale Policlinico San Martino*, Genova, Italy, using the patient's identity code (pseudonym) from countries governed by the General Data Protection Regulation (GDPR) (i.e., European countries). After a patient's eCRF is finalized and approved by the local investigator, it is fully anonymized or pseudonymized according to local practice and regulations.

### Data to be collected

This study is divided into three ‘modules’, according to the different aims described above:

- The BASIC module: to capture basic information on rehabilitation practice worldwide, and simple ICU and hospital outcomes, including length of stay in ICU and hospital; duration of ventilation and weaning; airway management; ICU complications, and death.
- The EXTENDED module: to capture granular data on type, timing, and duration of rehabilitative interventions, who delivers them, and safety assessment. In this module, we focus on the following rehabilitation interventions: passive interventions (passive exercises, stretching, cycling, electrical muscle stimulation, continuous passive motion); active interventions (exercise therapy, activities of daily living training, mobilization, cycling); respiratory physiotherapy (positioning, airways clearance techniques, lung expansion exercises, end-positive airway pressure devices, active cycle of breathing techniques, forced expiratory techniques, assisted or stimulated cough maneuvers, insufflation/ exsufflation, inhaled therapy); and adjunctive techniques (dysphagia training, swallow screening test, cognitive/ phycological therapy); and
- The EXTENDED FOLLOW-UP module: to capture quality of life and functional performance at 28 days after ICU discharge. Data collected to assess functional performance and quality of life are presented in the Items 2S - 5S (Supplementary Material).

Rehabilitation exposure will be operationalized at increasing levels of granularity according to module participation. In the basic module, exposure is captured as a binary variable (any rehabilitation vs none). In the extended module, exposure is further characterized by intervention type, timing from ICU admission, session duration, frequency, and level of patient participation (passive *versus* active).

The following data are collected: patient demographics, baseline characteristics, and details of rehabilitation practices. Definitions can be found in Item 6S (Supplementary Material). The following timeline outlines the processes and assessments involved from ICU admission through to discharge and the 28-day follow-up. Data collection flow-chart (Figure 1).

- ICU admission (Day Zero): patient baseline information & pre-hospital status; essential pre-hospital features including Simplified Acute Physiology Score (SAPS) II score; Acute Physiology and Chronic Health Evaluation (APACHE) II;^(33)^ Sequential Organ Failure Assessment (SOFA) score;^(34)^ invasive and noninvasive mechanical ventilation settings; sedation and muscle relaxants; vital signs; temperature; arterial blood gas analysis; delirium and pain assessment; hemodynamic monitoring; general rehabilitation assessment. Although REPOrt includes only patients receiving invasive mechanical ventilation, data on non-invasive respiratory support (e.g., non-invasive ventilation or continuous positive airway pressure) are collected when used during the weaning phase, after extubation, or as part of respiratory physiotherapy interventions.
- During ICU stay (Days Zero, 3, 7, 14): daily SOFA score, level of cooperation: S5Q scale;^(24,35)^ muscle strength using Medical Research Council (MRC) sum scale;^(36)^ ICU mobility scale;^(37)^ daily intervention duration; types of interventions: passive and active interventions; respiratory physiotherapy specifics (active and passive); occupational therapy, speech language therapists, other techniques applied; monitoring serious adverse events during interventions;
- ICU discharge: outcome assessment: patient pronation; ventilator weaning; airway management; monitor for any ICU complications; and
- 28-day follow-up after ICU discharge (day 28): post-discharge location (home, hospital, rehabilitation; assess return to work and daily activities; evaluate living arrangements - with family or alone); health assessments (swallowing disorders); tracheostomy cannula; home ventilator usage; clinical frailty scale; quality of life assessments using the EQ-5D-5L scale; and Modified Rankin Scale (mRS); mortality assessment.

**Figure 1:**
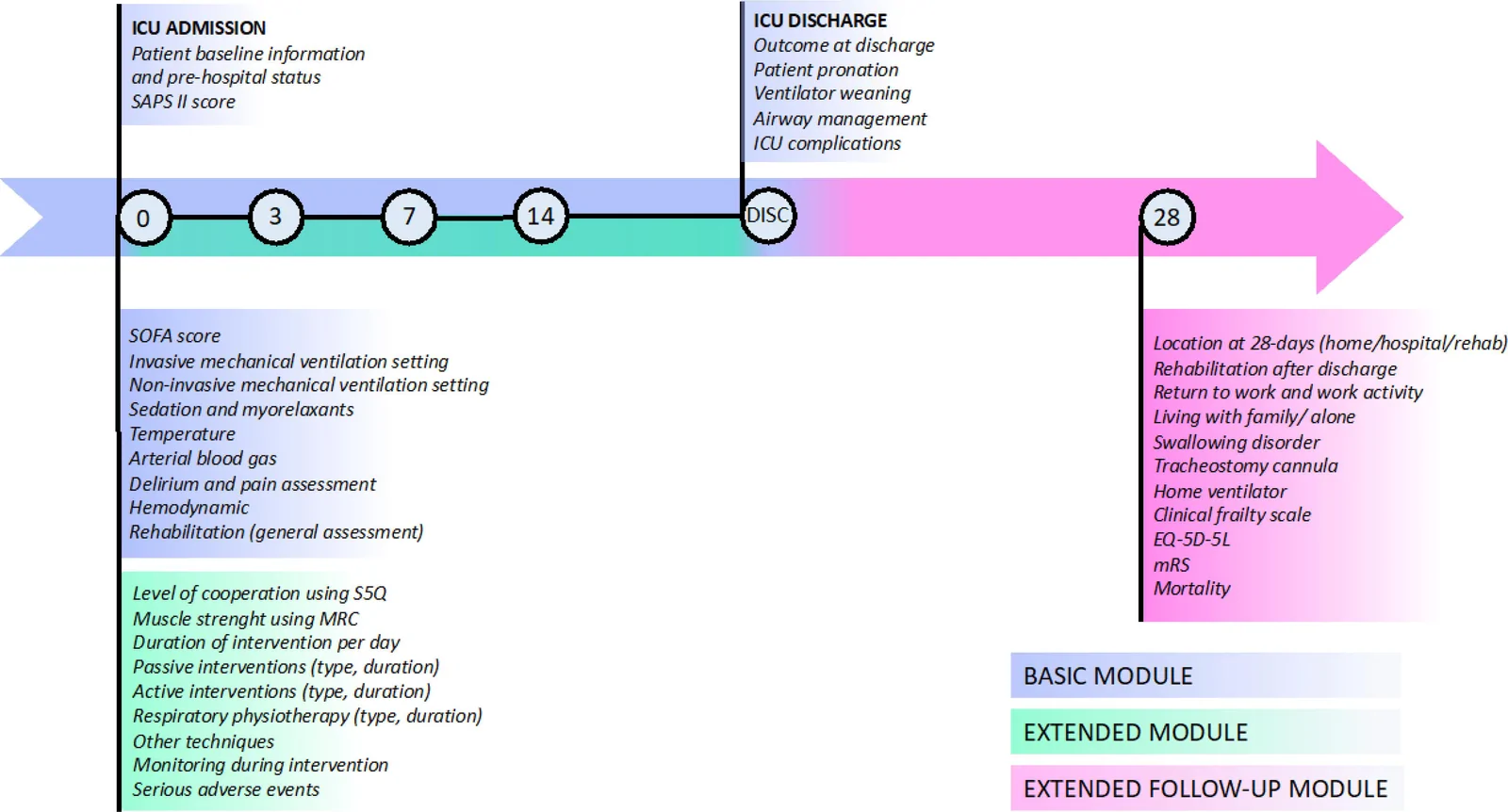
Study timeline from intensive care unit admission to 28-day follow-up.

ICU - intensive care unit; SAPS - Simplified Acute Physiology Score; DISC - discharge; SOFA - Sequential Organ Failure Assessment; mRS - modified Rankin score; S5Q - standardized five questions; EQ-5D-5L - mobility, self-care, usual activities, pain/discomfort, anxiety/depression; MRC - Medical Research Council.

Rehabilitation exposure is defined as any documented rehabilitation intervention during intensive care unit stay, with increasing detail captured in optional modules.

### Monitoring and reporting

The national coordinators will oversee the integrity of ethical approval in their country, the data collected, and adherence to standardized protocols and regulations, ensuring compliance with study protocols and maintaining data integrity. Regular communication will be established among ICUs to monitor enrolment progress and address any disparities. They will facilitate communication between ICUs and provide support for patient enrolment.

Each ICU will designate a lead investigator who will oversee local recruitment efforts and ensure accurate data collection. They will be asked to provide their ethical approval to the national coordinator, and they will also be responsible for reporting enrolment numbers and any challenges faced during the process.

### Sample size

Considering the exploratory nature of this global study and to ensure robust findings, the aim is to enroll approximately 2,400 patients across 60 participating ICUs. The sample size is based on an epidemiological assumption that around 40% of invasively ventilated patients receive rehabilitation. The assumed prevalence of 40% refers to the composite primary endpoint of any rehabilitation exposure and not to individual components such as passive or active mobilization alone. Considering a ± 2 margin of error, a minimum of 2,305 patients is required. Accounting for an anticipated 5% dropout rate, the target enrolment increases to 2,426 patients, which is rounded to 2,400 for practical purposes. This sample size will provide a comprehensive overview of current rehabilitation practices and their associated clinical outcomes in critically ill patients. This sample size is calculated to ensure adequate precision for descriptive prevalence estimates. It is not intended to provide formal statistical power for detecting associations between rehabilitation exposure and clinical outcomes.

### Statistical analysis plan

The primary analytical focus of REPOrt is descriptive. Analyses will quantify the prevalence, patterns, timing, and characteristics of rehabilitation across centers and regions. Secondary analyses exploring associations between rehabilitation exposure and outcomes will be considered exploratory and hypothesis-generating. Given the observational design, no causal inference is intended. Rehabilitation exposure will be defined without a priori minimum dose or frequency thresholds. If sufficient data are available, exploratory analyses may examine rehabilitation intensity and dose-response patterns using descriptive summaries and categorized exposure variables.

All patients meeting the inclusion criteria with available rehabilitation data will be included in the main analyses. Patients with complete data in the extended or extended follow-up modules will be analyzed separately.

Descriptive statistics will summarize patient characteristics, rehabilitation practices, and outcomes. Exploratory analyses of associations between rehabilitation and clinical outcomes may use regression methods appropriate to the outcome type, with adjustment for confounding and consideration of clustering as appropriate. To address confounding by indication, adjusted analyses will be guided by prespecified clinical domains rather than a fixed set of mandatory variables. These domains may include baseline patient characteristics, markers of acute illness severity and organ dysfunction, ICU clinical status, and functional status, when available and relevant to the outcome of interest. Clustering by center will be considered using approaches appropriate to the outcome and data structure.

Time-to-event outcomes, repeated measures, and follow-up assessments may be analyzed using suitable methods for their type and distribution. Time-varying covariates may be incorporated when feasible to reflect evolving clinical status. Sensitivity analyses may address missing data, model choices, or potential confounding.

All adjusted analyses will be interpreted as associative rather than causal. The extent and pattern of missing data will be described, and analyses will primarily rely on available data, with sensitivity analyses performed when missingness is substantial. All analytical decisions will be finalized before database lock and documented in a detailed statistical analysis plan to ensure transparency and reproducibility. Pre-planned secondary analyses are available in Item 7S (Supplementary Material). All analyses will be conducted using validated statistical software, with a conventional threshold for statistical significance, unless otherwise specified in an updated and finalized analysis plan.

## DISCUSSION

The proposed international observational study, REPOrt, aims to investigate rehabilitation practices in ICUs worldwide, focusing on critically ill patients on invasive ventilation who often develop ICUAW. This condition is associated with increased mortality and long-term morbidity.^(2-8)^ Rehabilitation interventions, including mobilization, physical activity, respiratory physiotherapy, and cognitive support, have shown benefits but remain underinvestigated.^(23,24,26,38)^

Research into the practice of rehabilitation in the ICU remains limited. Particularly, we lack global data on epidemiology, practice, timing, dosage, and intensity. The TEAM trial serves as an exemplary model of mobilization practices within a structured, protocolized framework and their impact on clinical outcomes. However, the challenge lies in replicating this approach effectively on a global scale to reach a broader audience. The variability in healthcare systems, resources, and patient populations across different regions raises important questions about the feasibility and adaptability of such protocols in diverse settings.^(30,32,39)^ Accordingly, REPOrt is primarily descriptive, with exploratory analyses intended to inform future interventional studies rather than to establish causal relationships.

Our protocol has several strengths. First, REPOrt looks beyond traditional rehabilitation methods, providing a comprehensive insight into various rehabilitation practices, both mobilization and respiratory physiotherapy, for invasively ventilated critically ill patients who are at higher risk of developing ICUAW.^(1)^ By examining diverse rehabilitation interventions such as mobilization, physical activity, respiratory physiotherapy, and cognitive support, REPOrt offers a holistic view of rehabilitation management in this patient population. Second, the study will be conducted as an international, multicenter, observational, multimodule investigation. Enrolling patients from diverse ICUs around the globe is a significant advantage. The inherent differences in practice, patient profiles, and treatment protocols create an opportunity to uncover geo-economic differences in rehabilitation practices and their associations with clinical outcomes. This broad scope enables stratified analyses, yielding more robust, representative data across regions. Third, REPOrt is designed specifically to minimize bias. Consecutive patients are enrolled using clear eligibility criteria to reduce selection bias. Data collection is standardized, with an eCRF designed to facilitate data collection, particularly in ICUs that may face challenges with traditional methods. Regular data audits will also be performed to mitigate information bias. Additionally, data will be collected on variables known to influence both exposure and outcomes, thereby reducing confounding bias. Moreover, the use of a multimodule structure allows for better adherence to data collection and quality standards, depending on resource availability.

Our study also has limitations. With participating ICUs across multiple countries and varying health record systems, data collection could prove challenging. The burden of data collection may be higher in some ICUs, especially those using paper records or that do not routinely collect the required information. To address this, REPOrt utilizes streamlined electronic forms, developed with input from collaborators in low-and middle-income countries, to facilitate data collection and minimize missing data. Moreover, substantial heterogeneity in rehabilitation expertise, staffing, and implementation is expected across participating centers. While this reflects real-world practice and is central to the study's objectives, it may contribute to variability in rehabilitation exposure and outcomes. Due to the observational nature of the design, this study cannot demonstrate causality but can only describe associations between rehabilitation practices and outcomes. Further randomized clinical trials would be necessary to establish causal relationships. Despite this limitation, the study aims to clarify the associations between rehabilitation interventions and outcomes, which are currently poorly understood. In addition, the requirement for at least 48 hours of invasive mechanical ventilation may introduce survivorship bias, as patients who die early or are rapidly extubated are not captured, potentially limiting generalizability to the sickest or most rapidly improving patients. Notably, the sample size was determined to support descriptive objectives rather than hypothesis testing; therefore, analyses of associations between rehabilitation and outcomes may be underpowered and should be interpreted cautiously. Confounding by indication remains an important limitation, as patients selected for rehabilitation are likely to be more stable and have a more favorable prognosis.

Although adjusted and time-updated analyses are planned, residual confounding cannot be fully excluded. Finally, differences in feasibility, consent procedures, and documentation across regions may result in center-level differential missingness. Such variability will be monitored and transparently reported to allow appropriate interpretation of findings. The success of REPOrt relies on active collaboration with participating ICUs. Data input will be conducted by local investigators, who will receive collaborative authorship on the final publication and may design sub-studies within the main protocol. This approach could introduce selection bias, as participating ICUs may have a heightened interest in rehabilitation, potentially making them less representative of the broader ICU population. To enhance ICU recruitment, REPOrt will seek endorsement from (inter)national bodies and societies focused on intensive care medicine.

## CONCLUSION

The findings of REPOrt have the potential to improve the care of invasively ventilated patients, as they may help identify global variation in rehabilitation practice and allow geo-economic comparisons. Last but not least, the findings of this study can be used for planning future studies.

## Data Availability

The contents are already available
