# Supplementary material for: Rehabilitation Practice and Association with Outcomes in Critically Ill Invasively Ventilated Patients (REPOrt): a protocol for an international multicenter observational study
Source: Crit Care Sci. 2026 Sep 2;38:e20260463. doi: 10.62675/2965-2774.20260463 (PMC13690032; doi:10.62675/2965-2774.20260463)
Supplement: Supplementary file 1 [file 2965-2774-ccsci-38-e20260463-suppl01.pdf]

Critical Care Science
https://doi.org/10.62675/2965-2774.20260463

SUPPLEMENTARY MATERIAL

Rehabilitation Practice and Association with Outcomes in Critically Ill Invasively Ventilated Patients (REPOrt): a protocol for an international multicenter observational study

Denise Battaglini[1], Rik Gosselink[2], Sabrine N. T. Hemmes[3], Carol L. Hodgson[4], Ricardo Kenji Nawa[5], Irene Schiavetti[6], Ary Serpa Neto[4], Gentle S. Shrestha[7], Marcus J. Schultz[8] for the REPOrt investigators*

ITEM 1S - ETHICAL CONSIDERATIONS

The REPOrt study will be conducted in accordance with the principles of the Declaration of Helsinki and the Medical Research Involving Human Subjects Act (WMO).[1] Data management, monitoring, and reporting of the study will be performed in accordance with the ICH-GCP Guidelines.

Approval to conduct the REPOrt at each participating center will be obtained from the local medical ethics committees. Since this study will be conducted as a multicenter study, the chief investigator at each participating site must obtain ethical approval from the local Ethics Committee to conduct the study in accordance with local regulations.

We anticipate that informed consent is not required for the basic and follow-up modules in most countries, as data are collected as part of routine care. The extended follow-up module, however, collects data that is not collected as part of routine care and requires informed consent in all countries. Written informed consent will be combined for participation in the basic and extended module, and separately for the extended follow-up module.

Of note, we will consistently adhere to local legislation on informed consent, including protocols for determining who may provide informed consent when a patient is deemed incapable of doing so themselves.

ITEM 2S - MUSCLE STRENGTH USING THE MEDICAL RESEARCH COUNCIL MUSCLE POWER SCALE

Muscle strength using the Medical Research Council (MRC) Muscle Power Scale (Grade 0 to 5):

- Grade 5: normal, movement against gravity and great/large resistance of the examiner.
- Grade 4: movement against gravity and moderate resistance of the examiner.
- Grade 3: movement against gravity over (almost) the full range of motion.
- Grade 2: movement of the limb with elimination of gravity.
- Grade 1: visible or palpable contraction without movement of the limb.
- Grade zero: no visible or palpable contraction.
- Shoulder abductors left and right.
- Elbow flexors, left and right.
- Wrist extensors left, right.
- Hip flexors left and right.
- Knee extensors, left and right.
- Foot dorsiflexors, left and right.

This is an open access article under the CC BY license (https://creativecommons.org/licenses/by/4.0/).

ITEM 3S - CLINICAL FRAILTY SCALE

- Very fit (regular exercise).
- Fit (occasionally active).
- Managing well (often are active, like walking).
- Very mild frailty (slowed up or tired during the day, no need for assistance).
- Mild frailty (evident slowing and need help with high order instrumental activities of daily living, like finances, transportation, housework).
- Moderate frailty (needing help with all outside activities, and with keeping house, frequently need help with stairs or with bathing).
- Severe frailty (completely dependent for personal care).
- Very severe frailty (completely dependent for personal care and approaching end of life).
- Terminally ill (life expectancy < 6 months).

ITEM 4S - EQ-5D-5L QUALITY OF LIFE QUESTIONNAIRE

We would like to know how bad your health is today. The scale is numbered from zero to 100, where 100 is the best health you can imagine, and zero the worst health you can imagine. Mark an X on the scale to indicate how your health is today.

Please write the number (from zero to 100) you marked on the scale here (________).

- Mobility
  - I have no problems walking about.
  - I have slight problems walking about.
  - I have moderate problems walking about.
  - I have severe problems walking about I am unable to walk about.
- Self-care
  - I have no problems washing or dressing myself.
  - I have slight problems washing or dressing myself.
  - I have moderate problems washing or dressing myself.
  - I have severe problems washing or dressing myself.
  - I am unable to wash or dress myself.
- Usual activities (e.g., work, study, housework, family or leisure activities)
  - I have no problems doing my usual activities.
  - I have slight problems doing my usual activities.
  - I have moderate problems doing my usual activities.
  - I have severe problems doing my usual activities.
  - I am unable to do my usual activities.
- Pain/ discomfort
  - I have no pain or discomfort.
  - I have slight pain or discomfort.
  - I have moderate pain or discomfort.
  - I have severe pain or discomfort.
  - I have extreme pain or discomfort.

- Anxiety/depression
  - I am not anxious or depressed.
  - I am slightly anxious or depressed.
  - I am moderately anxious or depressed.
  - I am severely anxious or depressed.
  - I am extremely anxious or depressed.

ITEM 5S - MODIFIED RANKIN SCALE (MRS)

Modified Rankin Scale:

- Zero: no symptoms at all.
- 1: no significant disability despite symptoms; able to carry out all usual duties and activities.
- 2: slight disability; unable to carry out all previous activities, but able to look after own affairs without assistance.
- 3: moderate disability; requiring some help, but able to walk without assistance.
- 4: moderately severe disability; unable to walk without assistance and unable to attend to own bodily needs without assistance.
- 5: severe disability; bedridden, incontinent, and requiring constant nursing care and attention.
- 6: dead.

ITEM 6S - DEFINITIONS

Ventilator-free days are the time between the first and last ventilation days, excluding patients who are no longer ventilated, either with a tracheostomy or a tracheostomy.

Extubation success is defined as the need for reintubation within hours or days after planned extubation.[2]

Ventilator-associated pneumonia (VAP) is defined as a lung infection that develops after 48 hours of mechanical ventilation.[3]

Ventilator-associated tracheobronchitis (VAT), defined as tracheobronchitis in a patient on a ventilator.[3]

Deep venous thrombosis is defined as a blood clot (thrombus) formed in one or more of the deep veins in the body.

Cardiovascular complications are defined as any of the following: coronary artery disease, peripheral arterial disease, or aortic disease.

Acute Respiratory Distress Syndrome (ARDS), defined according to the 2024 New Global definition as: (1) presence of acute hypoxemic respiratory failure, (2) onset within 7 days of insult, or new (within 7 days) or worsening respiratory symptoms; (3) bilateral opacities on chest x-ray or computed tomography (CT) or lung ultrasound not fully explained by effusions, lobar or lung collapse, or nodules; and (4) cardiac failure not the primary cause of acute respiratory failure. The following categories will apply: (1) Non-intubated ARDS: partial pressure of oxygen (PaO_2)/ fraction of inspired oxygen (FiO_2) ≤ 300mmHg or peripheral capillary oxygen saturation (SpO_2)/FiO_2 ≤ 315mmHg (if SpO_2 ≤ 97%) on high flow nasal oxygen with flow ≥ 30 L/min or non-invasive mechanical ventilation (NIV)/ continuous positive airway pressure (CPAP) with at least 5 cmH_2O positive end-expiratory pressure (PEEP); (2) Intubated ARDS: Mild - 200 < PaO_2/FiO_2 ≥ 300mmHg or 235 < SpO_2/FiO_2 (if SpO_2 ≤ 97%); Moderate - 100 < PaO_2/FiO_2 ≤ 200mmHg or 148 < SpO_2/FiO_2 ≤ 235mmHg (if SpO_2 ≤ 97%); Severe - PaO_2/FiO_2 ≤ 100mmHg or ≤ 200mmHg or SpO_2/FiO_2 ≤ 148mmHg (if SpO_2 ≤ 97%). Modified definition in Resource-Limited Settings: SpO_2/FiO_2 ≤ 315 (if SpO_2 ≤ 97%) not requiring PEEP or minimum flow rate of oxygen.[4]

Acute hypoxemic respiratory failure (non-ARDS), defined as PaO_2 < 60mmHg.[5]

Hypercapnic respiratory failure is defined as $PaCO_2$ > 45mmHg with a pH < 7.35 due to respiratory pump failure and/or increased CO_2 production.[5]

Deep venous thrombosis is defined as a blood clot (thrombus) formed in one or more of the deep veins in the body.[6]

Cardiovascular complications are defined as any of the following: coronary artery disease, peripheral arterial disease, or aortic disease.[7]

Number of days that the patients are alive and out of the hospital at 28 days after initiation of physiotherapy and rehabilitative interventions.

Quality of Life (QoL) will be evaluated using the EQ-5D-5L score, physical activity, and cognitive outcomes at 28 days.[8]

Safety assessment, defined as an event immediately related to rehabilitation/ physiotherapy practice:

- Falls out of the bed.
- Accidental removal of lines and catheters.
- Arrhythmias (tachycardia - bradycardia).
- Cardiac arrest.
- Noradrenaline/adrenaline infusion rate has increased by more than 25% during the rehabilitation. intervention compared to the start.
- Need for increasing FiO_2 more than 20% or any increase in PEEP.
- Major desaturation (SpO_2 < 80% for more than 1 minute).
- Unplanned extubation.

ITEM 7S - PREPLANNED SECONDARY ANALYSES

Preplanned secondary analyses include:

- Impact of rehabilitation on respiratory complications.
- Impact of rehabilitation on tracheostomy and weaning outcomes.
- Impact of sedation and delirium on rehabilitation practice and outcomes.
- Practice of rehabilitation in neurocritically ill patients.
- Impact of rehabilitation on invasive and non-invasive mechanical ventilation practice.
- Cost-effectiveness analysis.

REFERENCES

1. General Assembly of the World Medical Association. World Medical Association Declaration of Helsinki: ethical principles for medical research involving human subjects. J Am Coll Dent. 2014;81(3):14-8.
2. Thille AW, Richard JC, Brochard L. The decision to extubate in the intensive care unit. Am J Respir Crit Care Med. 2013;187(12):1294-302.
3. Torres A, Niederman MS, Chastre J, Ewig S, Fernandez-Vandellos P, Hanberger H, et al. International ERS/ESICM/ESCMID/ALAT guidelines for the management of hospital-acquired pneumonia and ventilator-associated pneumonia: Guidelines for the management of hospital-acquired pneumonia (HAP)/ventilator-associated pneumonia (VAP) of the European Respiratory Society (ERS), European Society of Intensive Care Medicine (ESICM), European Society of Clinical Microbiology and Infectious Diseases (ESCMID) and Asociación Latinoamericana del Tórax (ALAT). Eur Respir J. 2017;50(3):1700582.
4. Matthay MA, Arabi Y, Arroliga AC, Bernard G, Bersten AD, Brochard LJ, et al. A New Global Definition of Acute Respiratory Distress Syndrome. Am J Respir Crit Care Med. 2024;209(1):37-47.
5. Mirabile VS, Shebi E, Sankari A, Burns B. Respiratory Failure in Adults. In: StatPearls. Treasure Island (FL): StatPearls Publishing; 2024.
6. Kakkos SK, Gohel M, Baekgaard N, Bauersachs R, Bellmunt-Montoya S, Black SA, et al.; Esvs Guidelines Committee. Editor's Choice - European Society for Vascular Surgery (ESVS) 2021 Clinical Practice Guidelines on the Management of Venous Thrombosis. Eur J Vasc Endovasc Surg. 2021;61(1):9-82.
7. Isselbacher EM, Preventza O, Hamilton Black J 3rd, Augoustides JG, Beck AW, Bolen MA, et al.; Peer Review Committee Members. 2022 ACC/AHA Guideline for the Diagnosis and Management of Aortic Disease: A Report of the American Heart Association/American College of Cardiology Joint Committee on Clinical Practice Guidelines. Circulation. 2022;146(24):e334-482.
8. Herdman M, Gudex C, Lloyd A, Janssen M, Kind P, Parkin D, et al. Development and preliminary testing of the new five-level version of EQ-5D (EQ-5D-5L). Qual Life Res. 2011;20(10):1727-36.
